# BETi enhance ATGL expression and its lipase activity to exert their antitumoral effects in triple-negative breast cancer (TNBC) cells

**DOI:** 10.1186/s13046-022-02571-3

**Published:** 2023-01-06

**Authors:** Teresa Rossi, Raffaella Zamponi, Mattea Chirico, Maria Elena Pisanu, Egidio Iorio, Federica Torricelli, Mila Gugnoni, Alessia Ciarrocchi, Mariaelena Pistoni

**Affiliations:** 1 Laboratory of Translational Research, Azienda USL-IRCCS di Reggio Emilia, 42123 Reggio Emilia, RE Italy; 2 High Resolution NMR Unit, Core Facilities, Istituto Superiore Di Sanità, 00161 Rome, Italy

**Keywords:** ATGL, BET inhibitors, Lipid metabolism, Triple-negative breast cancer

## Abstract

**Background:**

Triple-Negative Breast Cancer (TNBC) is a subtype of breast cancer that differs from other types of breast cancers in the faster spread and worse outcome. TNBC presented limited treatment options. BET (Bromodomain and extra-terminal domain) proteins are epigenetic readers that control the expression of different oncogenic proteins, and their inhibition (BETi) is considered a promising anti-cancer strategy. Recent evidence demonstrated the involvement of BET proteins in regulation of metabolic processes.

**Methods:**

MDA-MB231 cells treated with JQ1 followed by RNA-sequencing analysis showed altered expression of lipid metabolic genes; among these, we focused on ATGL, a lipase required for efficient mobilization of triglyceride. Different in vitro approaches were performed to validate the RNA-sequencing data (qRT-PCR, immunofluorescence and flow cytometry). NMR (Nuclear Magnetic Resonance) was used to analyze the lipid reprogramming upon treatment. ATGL expression was determined by immunoblot and qRT-PCR, and the impact of ATGL function or protein knockdown, alone and in combination with BETi, was assessed by analyzing cell proliferation, mitochondrial function, and metabolic activity in TNBC and non-TNBC cells culture models.

**Results:**

TNBC cells treated with two BETi markedly increased ATGL expression and lipolytic function and decreased intracellular lipid content in a dose and time-dependent manner. The intracellular composition of fatty acids (FAs) after BETi treatment reflected a significant reduction in neutral lipids. The short-chain FA propionate entered directly into the mitochondria mimicking ATGL activity. ATGL KD (knockdown) modulated the levels of SOD1 and CPT1a decreasing ROS and helped to downregulate the expression of mitochondrial ß-oxidation genes in favor of the upregulation of glycolytic markers. The enhanced glycolysis is reflected by the increased of the mitochondrial activity (MTT assay). Finally, we found that after BETi treatment, the FoxO1 protein is upregulated and binds to the PNPLA2 promoter leading to the induction of ATGL. However, FoxO1 only partially prompted the induction of ATGL expression by BETi.

**Conclusions:**

The anti-proliferative effect achieved by BETi is helped by ATGL mediating lipolysis. This study showed that BETi altered the mitochondrial dynamics taking advantage of ATGL function to induce cell cycle arrest and cell death.

**Graphical Abstract:**

Schematic representation of BETi mechanism of action on ATGL in TNBC cells. BETi induce the expression of FoxO1 and ATGL, lowering the expression of G0G2, leading to a switch in metabolic status. The induced expression of ATGL leads to increased lipolysis and a decrease in lipid droplet content and bioavailability of neutral lipid. At the same time, the mitochondria are enriched with fatty acids. This cellular status inhibits cell proliferation and increases ROS production and mitochondrial stress. Interfering for ATGL expression, the oxidative phenotypic status mildly reverted to a glycolytic status where neutral lipids are stored into lipid droplets with a consequent reduction of oxidative stress in the mitochondrial.
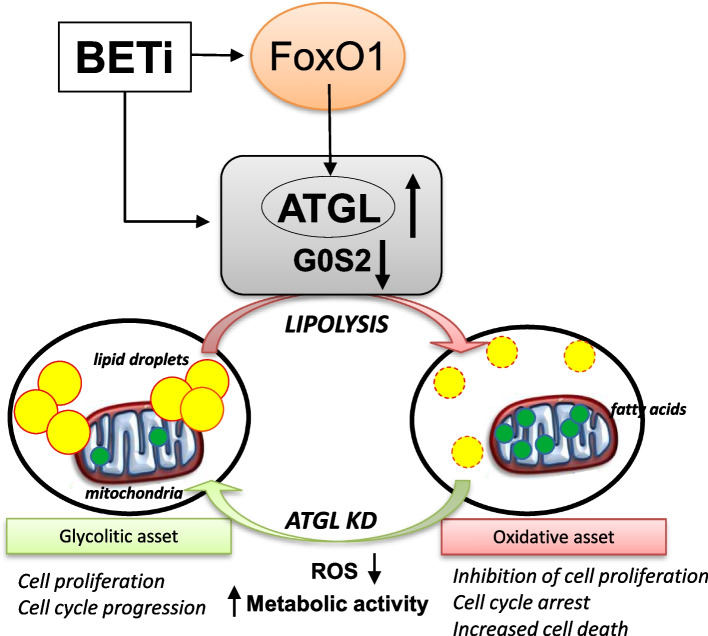

**Supplementary Information:**

The online version contains supplementary material available at 10.1186/s13046-022-02571-3.

## Background

Triple-negative breast cancers (TNBC) account for about 10–15% of all breast cancers (BC). Unlike the other invasive breast cancers (BC), TNBC grows and spreads faster, with a worse prognosis and limited treatment options. For this reason, the challenge is to seek new strategies capable of limiting the aggressiveness of the TNBC. Cancer requires major metabolic modifications to sustain its growth [1] and breast tumors are not excluded from these metabolic modifications. Tumor progression is accompanied by changes in cellular metabolism to meet increasing demands for energy, biomass, and redox maintenance [2]. The crucial role of glucose metabolism in TNBC and other cancer types is known and its withdrawal can induce cell death. However, emerging evidence suggests that the alterations in fatty acids (FAs) synthesis, oxidation (FAO), and phospho- and neutral lipid metabolism contribute to rapid tumor growth [1, 3–6]. It has been shown that in TNBC, both FAs synthesis and oxidation are upregulated [7]. FAs are described as essential biomolecules [8] and are considered necessary energy resources whose oxidation generates the highest energy yield for ATP. However, when their intra- and/or extracellular concentrations exceed the physiological levels, they become toxic to the cell (lipotoxicity). For this reason, lipid droplets (LDs) play a vital role in storing excess FAs as neutral lipids, mostly triacylglycerol (TAG) and cholesterol esters and releasing them under stress conditions for cancer cell survival [9, 10]. LDs are dynamic organelles believed to function as cytoplasmic storage depots with main functions in lipid and energy homeostasis [11]. Higher accumulation of LDs, found in several cancer cells, including BC, have been associated with tumor aggressiveness and chemotherapy resistance [12]. Lipolysis controls the lipid content and mobilization of FAs stored in the LDs with the sequential breakdown of TAG into FAs and glycerol. The enzyme responsible for the first step of TAG hydrolysis is the adipose triglyceride lipase (ATGL, encoded by PNPLA2-Patatin Like Phospholipase Domain Containing 2) [13–15]. ATGL is localized at LDs surface, and alterations in its expression and/or function affect a plethora of FA-dependent processes, including oxidative activity and lipid accumulation in various tissues. Mounting evidence demonstrated how genetic and epigenetic alterations can induce cell-selective metabolic changes [16]. BRD4, a transcriptional and epigenetic regulator that plays a pivotal role in cancer, is a member of the Bromodomains and Extraterminal (BET) family of chromatin regulators, a family of proteins that also comprised BRD2, BRD3, and BRDt [17, 18]. BET proteins’ function is hindered by BET inhibitors (BETi), which induce growth inhibition in several tumors, and they have been regarded as promising anticancer strategies [19, 20]. Recent studies have showed that BET proteins are also involved in regulating the metabolic process. For instance, BETi strongly affect autophagy induction, reactive oxygen species (ROS) production, and glucose metabolism. Conversely, the role exerted by BET proteins on lipid metabolism is still poorly characterized [21]. Despite little evidence, no studies systematically addressed the involvement of BET proteins in the modulation of proteins and enzymes controlling the regulatory machinery of lipid metabolism. Thus, we aim to evaluate if BETi can rewire cell metabolism releasing FAs from the LDs and reducing cell growth by inducing cell lipotoxicity. A better understanding of this mechanism is essential to select innovative therapeutic targets, which might be a promising strategy to restrain cancer aggressiveness.

## Methods

### Cell lines, transfections, and treatments

Human breast cancer cell lines MDA-MB231 (referred to as MB231, human triple-negative metastatic) and Hs578T (human triple-negative primary) were purchased from ATCC. MCF7 (human breast cancer luminal) was purchased from Sigma-Aldrich. BT549 (human triple-negative primary) cell line was a kind gift from Dott.ssa Paola Bonetti at IFOM-IEO campus, Milan, Italy. Cell lines were authenticated by SNP profiling at Multiplexion GmbH, Germany. MDA-MB231 and BT549 cell lines were grown at 37 °C/5% CO2 in DMEM with 10% FBS (ThermoFisher scientific). Hs578T was grown at 37 °C/5% CO2 in DMEM (Invitrogen) medium with 10% FBS and 0.01 mg/ml human insulin (Sigma-Aldrich). MCF7 were grown at 37 °C/5% CO2 in MEM + GlutaMAX (plus Earle’s Salts, Invitrogen) medium with 10% FBS and 1% of NEAA (Invitrogen). All media were supplemented with 1% penicillin-streptomycin (Life Technologies). All cell lines were tested monthly for Mycoplasma contamination. Select small interfering RNA (siRNA) against PNPLA2 (ID: s32682, siATGL), FoxO1 (ID: s25258, siFoxO1), and negative control oligos (siCNT) (Ambion) were transfected using 25 nmol/L of siRNA with RNAiMax Lipofectamine (Thermo Fisher Scientific) reagent according with the reverse transfection protocol. Cells were harvested and analyzed 48 and 72 hours after transfection. BET inhibitors were added to siATGL, siFoxO1, and siCNT 24 h after the transfection at the time and doses indicated. Flag-FOXO1A plasmid was a gift from Stefan Koch (Addgene plasmid # 153141; http://n2t.net/addgene:153141; RRID:Addgene_153141) and was transfected with Lipofectamine2000 (Thermo Fisher Scientific) reagent according with the transfection/manufacturer’s protocol. For drug treatment, cells were plated at sub confluence density 24 hours before treatment. DMSO (referred to as CNT, Sigma-Aldrich) stocks of JQ1, OTX015, and ATGListatin (all purchased from Sigma-Aldrich) were diluted in the cell culture medium at the time and indicated concentrations. Propionic acid (PA, 402907) was purchased from Sigma-Aldrich. Oleic acid (OA) was a kind gift from Dott. Egidio Iorio, Istituto Superiore di Sanità, Rome, Italy. Fatty acids (FAs) were added to the culture medium at the indicated concentrations.

### BrdU incorporation assay

In order to avoid cell–cell-contact-induced inhibition of cell proliferation due to cell confluency, we seeded 3-5 × 10^5^ cells per well in 6 wells plate. This procedure ensured that cells were in a sub confluent state after 1 day and 3 days of incubation. 10 μM of 5-bromo-2′-deoxyruridine (BrdU, Sigma-Aldrich) was added to cell cultures for 1 hour. After 1 hour pulse, the cells were fixed in ice-cold 70% ethanol dropwise on a vortex and left at 4 °C for 30 minutes, washed twice with PBS and suspended in 2 M HCl for 30 minutes at room temperature (RT) with occasional mixing. The cells were subsequently washed twice in PBS and 2.5ul of anti-BrdU mAb (B44, BD biosciences) in BSA-PBS-Tween (PBS + 0.1% BSA + 0.2% Tween20, pH 7.4) were added to the cell pellet at RT for 30 minutes in the dark. After washing the cells twice, 2.5ul of goat anti-mouse FITC (BD555988, goat anti mouse) in PBS-Tween was added to the cells at RT for 30 minutes. The pellet was washed in PBS, 10μg/ml of RNAse (Ribonuclease A, R4642, Sigma-Aldrich) was added for 15 minutes at 37 °C and 50μg/ml of PI (Propidium Iodide, Sigma-Aldrich, #P4170) was added prior flow cytometry analysis. We collected at least 30,000 events at the BD FACS Canto II (BD Biosciences).

### Cell death and CFSE detection

Following the protocol’s instruction, cell death was quantified using FITC Annexin V Apoptosis detection Kit (BD Biosciences, #559763). Briefly, 5 × 10^5^ cells/well were plated in a 6-well plate, and both supernatant and attached cells were used for the analysis. Cells were washed with PBS, resuspended in Annexin V-binding buffer (1X in water). Cells were stained with Annexin V-FITC (Fluorescein isothiocyanate) and 7-amino-actinomycin D (7-AAD) for 15 min at + 4 °C in the dark. Analysis was performed by flow cytometry using a FACSCanto II (BD Biosciences) on FSC/SSC viable gated cells, excluding cell debris and doublets. Samples were analyzed using FACSDiva Software.

For cell duplication rate analysis, we used a CFSE (5(6)-Carboxyfluorescein N-hydroxysuccinimidylester) staining by CFSE - Cell Labeling Kit (Abcam, #ab113853) following manufacture’s protocol. Briefly 5 × 10^4^ cells/condition were washed with PBS and resuspended in PBS/CFSE (4 μM) for 15 minutes at 37 °C in the dark. The reaction was quenched by adding an equal volume of medium for 5 minutes. The cells were washed to remove unincorporated CFSE and immediately after that After that, plated and analysis was every 24 hours by flow cytometry using a FACSCanto (BD Biosciences). Samples were analyzed using FACSDiva Software.

### Cell proliferation and Colony-forming assays

For BETi efficacy, a proliferation assay was performed, plating 5 × 10^3^ cells per well in 96-wells, and viable cells were counted 72 hours after BETi treatment using trypan blue (Sigma-Aldrich) staining and the Bürker chamber for the count. Proliferation analysis with FAs and ATGListatin were performed, seeding 2 × 10^3^ cells per well in 96-wells. The day after, cells were treated and placed in IncuCyte S3 Live-Cell Analysis System (Essen BioScience). Cell proliferation was monitored for 96 hours, and images were collected every 12 hours using the phase-contrast confluence metric. For the colony formation assay, cells were seeded in 6-wells plates at a density of 500–600 MDA-MB231 cells/well and 1000–1200 Hs578T cells/well and cultured for 10–12 days. Then, colonies were fixed with methanol, stained with 0.5% Crystal Violet (Sigma-Aldrich) and counted with ImageJ software [22].

### Lipid staining

Oil Red O (ORO) working solution (Sigma-Aldrich O-06525, ORO stock solution 0.3% in isopropanol) was prepared by diluting 6 parts of ORO stock with 4 parts of water. 3 × 10^5^ cells were seeded in 12-well plates. After 24 hours of BETi treatment, cells were fixed in paraformaldehyde (PFA-4% solution, Sigma-Aldrich) for 10 minutes and rinsed twice with PBS. Each well was washed with 60% isopropanol (Sigma-Aldrich) and let dry completely. Next, cells were stained with 0.5 ml/well of ORO for 10 minutes at room temperature. Subsequently, wells were rinsed under running tap water until no excess stain was seen. 1 ml of hematoxylin was added into each well to counterstain the nuclei for 1 minute. Finally, wells were washed until no excess stain was seen and viewed on a phase-contrast microscope (Nikon). Lipid appeared red and nuclei blue.

BODIPY™ 500/510 C1, C12 (4,4-Difluoro-5-Methyl-4-Bora-3a,4a-Diaza-s-Indacene-3-Dodecanoic Acid, ThermoFisher scientific) stock solution, 1 mg/mL, was prepared in ethanol (Sigma-Aldrich) and kept at − 20 °C until used. Cells were incubated overnight with 1 μg/mL BODIPY™ in co-administration with the specific treatment.

### Immunofluorescence (IF) and flow cytometry assays

For IF, 6 × 10^4^ cells were seeded onto chambers slide (4 chambers, Eppendorf), washed with PBS, and fixed with 4% paraformaldehyde for 10 minutes. For BODIPY staining only, the cells were permeabilized with 0.1% Triton (Sigma-Aldrich) in PBS for 5 minutes, washed with PBS, and stained with DAPI (Invitrogen). For ATGL staining, the cells were permeabilized 10 minutes with 0.1% Triton, washed with PBS, blocked 1 h in 2% BSA/PBS and stained with 1:100 ATGL antibody (Invitrogen, PA5–17436) in 0.1% BSA (Sigma-Aldrich)/PBS overnight at 4 °C. The day after, cells were stained with secondary antibody anti-rabbit Alexa 594 (Thermo Fisher Scientific) and DAPI. Coverslips were mounted with Slowfade long antifade Mountant (Invitrogen). Immunofluorescences were detected using confocal microscopy (LEICA).

For flow cytometry, cells were seeded and treated with specific drugs for 24 hours, and BODIPY was added overnight. Afterward, cells were washed with PBS, detached, and resuspended in PBS with 3 mM EDTA, and analyzed for at least 10,000 events at the BD FACS Canto II (BD Biosciences). Median Fluorescence Intensity (MFI) was calculated by setting the gate at 50% of the CNT and analyzing the curve’s shift of the treated cells.

### RNA isolation and reverse-transcription quantitative PCR assays

Total RNA for gene expression was extracted with Maxwell RSC simplyRNA Cells (Promega). Up to 1 μg of RNA was synthesized to cDNA using the iScript cDNA Kit (Bio-Rad). Reverse-transcription quantitative PCR (RT-qPCR) was conducted using Sso Fast EvaGreen SuperMix (Bio-Rad) and primers mixed at a final concentration of 250 nmol/L in the CFX96 Real-Time PCR Detection System (Bio-Rad). Fold change was calculated by the 2^(−ΔΔCt)^ method. βACTIN (b-ACT) was used as a housekeeping gene. RT-qPCR primers are listed in Table 1.

**Table 1:** Primers used in this study

Gene Name	Forward	Reverse
**ACER2**	GTAGGTTCAAGGTGGTGGTCAG	CACACGCATGTTGTCACACCT
**ACACB**	CACAGGTGAAGCTGAGACCC	CCCCAAAGCGTGTGACAAAC
**PNPLA2**	CAACGCCACGCACATCTAC	GCTTCCGGGCCTCTTTAGAT
**DGAT1**	CCCCAACAAGGACGGAGAC	TGAAGCCACTGTCAGAGCTG
**SMPD3**	CTACCCCATCATGGACGTGG	GCGATGTACCCGACGATTCT
**SYNJ1**	TCAATCAGAAAAATGCTGGACA	GGCACTTTTCACTGGTGTCA
**MTM1**	AAGACCCAGCGTAAATGCAG	AAGATTCCCGCATAACATGAA
**ACSL1**	AGTGAGCCTGTTGCTCAGGT	CTGCACAGTTCCTCAAACGA
**SC5D**	TGCACTGTTCTTGCTGGAGA	TGAAGGCCTCTGTGAATCCAG
**SLC16A1/MCT1**	GGCTTGATTGCAGCTTCTTT	CAATCATGGTCAGAGCTGGA
**SLC16A4/MCT4**	AAGAGCCATTGTATGTCTGGG	GGGACTTGCCAGTTTCTTTG
**PKM2**	TCGCATGCAGCACCTGATT	CCTCGAATAGCTGCAAGTGGTA
**SLC2A1/GLUT1**	TTCACTGTCGTGTCGCTGTT	TGAGTATGGCACAACCCGC
**SLC2A3/GLUT3**	GGACGTGGAGAAAACTTGCTG	TCAGAGCTGGGGTGACCTTC
**SLC2A4/GLUT4**	AGCACCGCAGAGAACACAG	GTCGGGCTTCCAACAGATAG
**ACAA2**	GTCTGCTGGCAAAGTCTCACC	ATTCCCACACGCAAACCAA
**ACADVL**	CAGGAGCCGTGAAGGAGAAG	GGCACCCGTACTCCATCAAA
**ECHS1**	GCCTCGGGTGCTAACTTTGA	GCCATCGCAAAGTGCATTGA
**ACADS**	CCTCAGCGAACCAGGGAAC	TTCAGAACCCATGAGTCGCC
**G0S2**	GGCCTGATGGAGACTGTGTG	CTTGCTTCTGGAGAGCCTGT
**FOXO1**	GAGGGTTAGTGAGCAGGTTACA	ACTGCTTCTCTCAGTTCCTGC
**CDKN1A (p21)**	GCCTGGACTGTTTTCTCTCG	ATTCAGATGTGGGAGGAG
**PIK3R1**	GCCATTGAAAAGAAAGGTCTGGA	TGAAAGCGTCAGCCAAAACG
**TGFb**	GCAGCACGTGGAGCTGTA	CAGCCGGTTGCTGAGGTA
**HOMER1**	GCGGGGATCTTCAGTCTCCT	TGCTGATTGCTGAACTATGTGA
**BCL6**	CTGCAGATGGAGCATGTTGT	TCTTCACGAGGAGGCTTGAT
**CCND2**	GGACATCCAACCCTACATGC	CGCACTTCTGTTCCTCACAG
**SIRT1**	AGGCCACGGATAGGTCCAT	CTCAGGTGGAGGTATTGTTTCC
**GADD45A**	CTGGAGGAAGTGCTCAGCAAAG	AGAGCCACATCTCTGTCGTCGT
**KLF2**	CACGCACACAGGTGAGAA	ACAGATGGCACTGGAATGG
**FOXO3**	CCTACTTCAAGGATAAGGGCGACAG	GTGCCGGATGGAGTTCTTCCAG
**BNIP3**	CGGGATGCAGGAGGAGAG	TAGAAACCGAGGCTGGAACG
**b-ACTIN**	TGCGTTACACCCTTTCTTGA	AAAGCCATGCCAATCTCATC

### RNA-sequencing (RNA-seq)

**Fig. 1:**
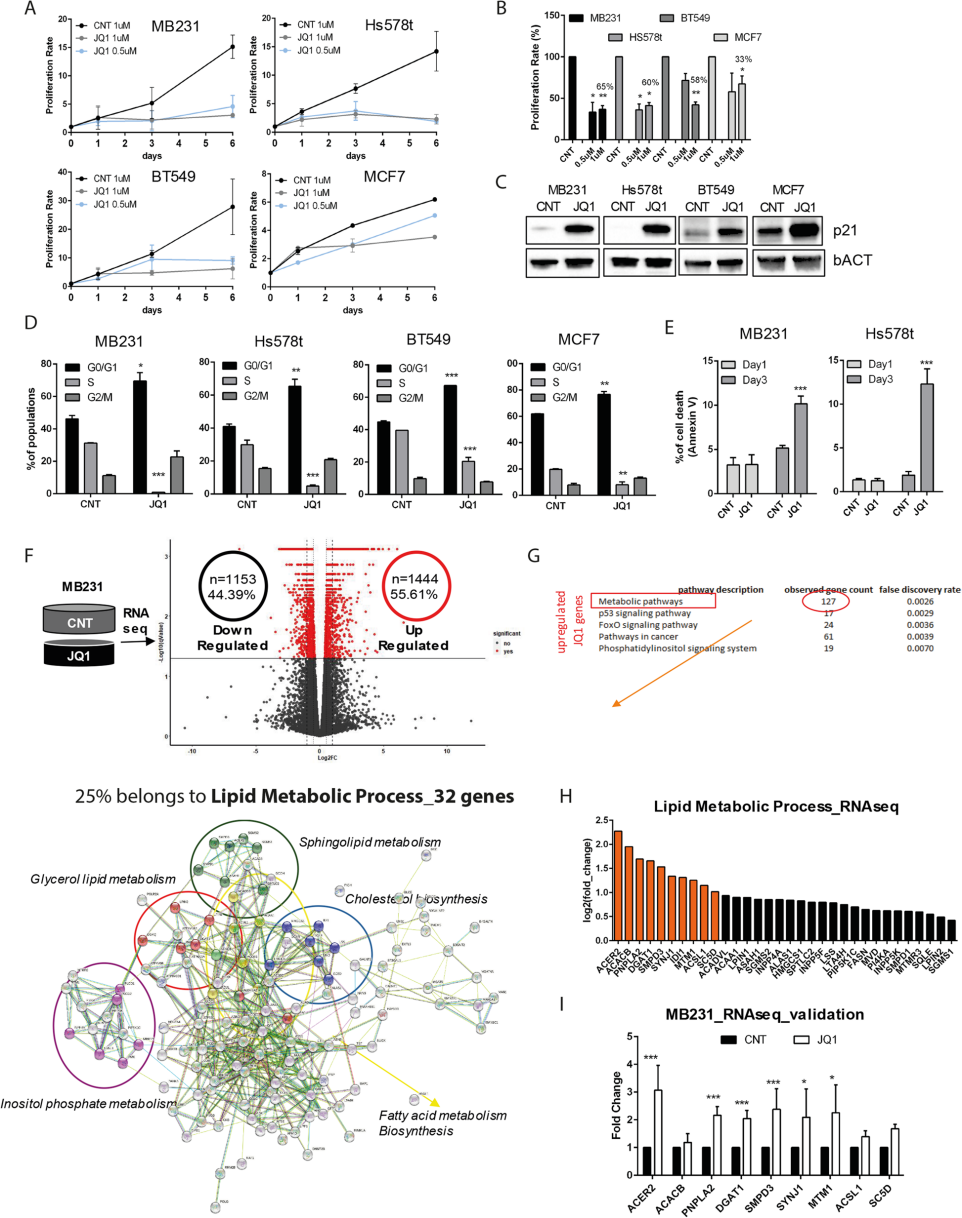
BET inhibitor JQ1 differentially affects the expression of genes involved in lipid metabolism. **A** Proliferation rate of MDA-MB231, Hs578t, BT549, and MCF7 cells with two doses of JQ1 (0,5 μM and 1 μM). **B** Proliferation rate at day 3 represented as a percentage of the cell number rate in A. **C** Immunoblotting for p21 in MDA-MB231, Hs578t, BT549 and MCF7 cells after treatment with CNT or JQ1 for 24 hours. bACT served as a loading control. **D** Cell cycle analysis of MDA-MB231, Hs578t, BT549 and MCF7 cells treated with 1 μM JQ1 for 24 hours. BrdU and PI incorporation indicated the % of population. Here, we plotted the % of cell population in all the three different phases of the cell cycle. The significance is calculated versus the CNT treated cells. **E** Percentage of cell death in MDA-MB231 and Hs578t cells treated with 1 μM JQ1 for 1 day (1d) or 3 days (3d) with Annexin V staining. The significance is calculated versus the CNT treated cells. **F** RNA-seq analysis of MDA-MB231 treated for 6 hours with 1 μM of JQ1. The volcano plot represents the differentially regulated genes (red = significant, black = not significant), on the right are the upregulated, while on the left are the downregulated genes. **G** Top 5 GO (gene ontology) categories of upregulated genes analyzed with STRING. The Metabolic pathway category denotes the 25% of genes directly related to lipid metabolism. On the STRING network, different colors discriminate the diverse lipid metabolic process category: in purple the Inositol phosphate metabolism; in blue the Cholesterol biosynthesis; in yellow the Fatty acid metabolism Biosynthesis; in red the Glycerol lipid metabolism and green the Sphingolipid metabolism. **H** Genes in the “lipid metabolic process” identified by RNA-seq plotted for the log2 fold change value. In orange, the genes were selected for further analysis with a log2 ≥ 1. **I** Validation of the selected genes in panel C by RT-qPCR analysis

RNA was extracted using TRIzol (Invitrogen) from MDA-MB231 treated with 1 μM of JQ1 for 6 hours. RNA-seq libraries were prepared using the TruSeq Stranded mRNA Sample Preparation Kit (Illumina), starting from 1 μg of RNA. RNA-seq was performed on NextSeq 500 platform (Illumina) using a dual strand 2 × 75 approach. A minimum of 20 million reads for each sample replicate were considered. In bioinformatics data analysis, Fastq quality check was carried on using FastQC, and adapters removal and sequence alignment was conducted by Trimmomatic and STAR, respectively. Cufflink RNA-Seq workflow was applied to proceed with reads count and normalization. Differential gene expression was calculated by the Cuffdiff pipeline as fold-change. Genes with a *P*-value< 0.05 were considered significantly deregulated. Data analysis was supported by using R software (version 3.4.3). We plot the genes in Fig. 1C based on log2 data with an arbitrary cutoff for the validation of log2 fold change ≥1.

### Lipidomic analysis

Deuterated reagents (methanol (CD3OD), chloroform (CDCl3)) and deuterium oxide (D2O) were purchased from Cambridge Isotope Laboratories, Inc.; 3-(trimethylsilyl) propionic-2,2,3,3-d4 acid sodium salt (TSP) was obtained from Merck & Co, Montreal, Canada. For the extraction of aqueous and organic metabolites, cell pellets were extracted according to the protocol previously described [23]. The polar phase containing water-soluble cellular metabolites was evaporated using a rotary evaporator and lyophilized while the organic fraction (lipid phase) was evaporated under nitrogen gas flow. Both phases of cell extracts were stored at − 20 °C. Lipid fraction from cells was resuspended in a CD3OD / CDCl3 solution (2:1 v/v) with 0.05%of tetramethylsilane (TMS) as internal reference. High-resolution 1H-NMR analyses were performed at 25 °C at 600 MHz (14 T Bruker AVANCE Neo spectrometer; Karlsruhe, Germany, Europe) on organic cell extracts using acquisition pulses, water pre-saturation, data processing, and peak area deconvolution as previously described [23]. Relative quantification of lipid signals in organic fractions was referred to the signal at 1.6 ppm (as a measure of total acyl chain and referred to 100). The integrals of characteristic lipid signals were compared to this value.

### Mitochondrial metabolic activity by MTT assay

Cells were seeded in a 96-wells plate at 3 × 10^3^ cells/well density. After siRNA transfection or drug treatment, 40 μl of a 5 mg/ml of MTT (1-(4,5-Dimethylthiazol-2-yl)-3,5-diphenylformazan, Thiazolyl blue formazan- Sigma-Aldrich) solution was added to each well. After 3 hours at 37 °C in the dark, the supernatant was discarded, and 100 μl of DMSO was added to dissolve the precipitate. The optical density of formazan was detected at 560 nm to estimate the metabolic activity with GloMax Discover Microplate Reader (Promega).

### ROS evaluation

Thirty minutes before the end of the experimental time (48 h of siATGL and siCNT transfection), cells were incubated with DHE (dihydroethidium) assay kit (Ab236206, Abcam) at 37 °C according to the manufacturer’s directions. Fluorescence was measured using the microplate reader (GloMax Discover Microplate Reader) at 520 nm (excitation) and 580–640 nm (emission). The fluorescence was normalized to the total number of cells.

### Chromatin immunoprecipitation

Chromatin immunoprecipitation (ChIP) was performed as previously described [24]. Briefly, chromatin was precipitated with FoxO1 antibody (rabbit mAb C29H4, Cell Signaling Technology) and the relative IgG control (rabbit IgG 2729S, Cell Signaling Technology). 1% of chromatin used for immunoprecipitation was kept as input control. Purified DNA was analyzed by RT-qPCR. Each RT-qPCR value was normalized over the appropriate input control and reported in graphs as a ratio over the IgG. The analyzed amplicon of the PNPLA2 genome mapped on FoxO1 binding site as highlighted by Jaspar TF tool in UCSC genome browser (Fig. 6D). Primers for PNPLA2 region for 5^′^- TTCATGGGTGAGGGTGCTTC − 3^′^ and rev: 5^′^- ACATCACTCCCTCATGGCAG − 3^′^.

### Western blot

Cells were lysed in Passive Lysis Buffer (PLB, Promega), resuspended in Laemmli 4x buffer (Bio-Rad), and boiled at 95 °C for 10 minutes. Total protein extracts were separated using an SDS-PAGE gel (Bio-Rad), transferred to nitrocellulose membrane (Bio-Rad) using the Trans-Blot Turbo Transfer System (Bio-Rad), and blocked 5% milk/PBS-0.1% Tween 20 (Sigma-Aldrich). The membranes were incubated with primary antibodies diluted in 2% BSA/PBS tween 0.1% overnight at 4 °C on a shaker. Primary antibodies used for the study were as follows: β-actin as a loading control (Sigma-Aldrich, A1978), p21 (Abcam, ab227443), ATGL (Invitrogen, PA5–17436), DGAT1 (SCBT, sc271934), SOD1 (Cell Signaling, 71G8), CPT1a (Abcam, ab128568) and FoxO1 (Cell Signaling, C29H4). Secondary antibodies were HRP-conjugated donkey anti-rabbit and sheep anti-mouse (GE Healthcare). Membranes were incubated with secondary HRP-conjugated antibodies for 1 hour at room temperature. Clarity ECL (Bio-Rad) or ECL prime (Amersham) were used for detection using ChemiDoc Imager (Bio-Rad) following the manufacturer’s instructions. ImageJ software was used to quantify the band intensity.

### Statistical analysis

Comparisons between two data groups were analyzed using an unpaired two-tailed Student t-test (GraphPad Prism 7). *P*-values < 0.05 were considered significant and indicated in the graphs by *. *P* values < 0.01 or < 0.001 were indicated by ** and ***, respectively. All data were shown as mean and SEM. Each experiment was replicated two to five times.

## Results

### JQ1 treatment affects the expression of genes involved in lipid metabolisms in MDA-MB231 cells

JQ1 antitumoral activity is mainly associated with its inhibitory on the BRD4 protein, whose expression is higher in TNBC cell lines than in other BC cell lines [25, 26]. BC cell lines were treated with different doses of JQ1 and we observed a reduction in cell number (Fig. 1A) of about 60% in TNBC cells compared to a 30% drop in the non-TNBC MCF7 cell line (Fig. 1B) already at day three. The growth arrest induced by JQ1 agreed with the up-regulation of p21^WAF1/CIP1^ (p21) in all BC cell lines (Fig. 1C). Flow cytometry analysis showed that all the BC cell lines have a significant decrease in the S-phase, with a concurrent increase of cells in the G0/G1 phase upon 24 hours of JQ1 treatment (Fig. 1D). We confirmed a decreased proliferation of MDA-MB231 and Hs578t cells after JQ1 treatment by CFSE assay (Supplementary Fig. 1A). The marked G0/G1-phase arrest upon JQ1 administration falls with the increased number of cells death after 3 days (Fig. 1E). Similar results were obtained with OTX015, another BET inhibitor currently in use in clinical trials for TNBC tumors [27] (data not shown). To investigate the early effect of JQ1 on TNBC cells and therefore exclude possible off-target effects, we performed RNA sequencing (RNA-seq) on MDA-MB231 cells treated with JQ1 for 6 hours. RNA-seq analysis showed that 2597 genes were differentially expressed (DEG) upon JQ1 treatment (> 2-fold change) with a comparable number of downregulated (*n* = 1153, 44.39%) and upregulated (*n* = 1444, 55.61%) genes (Fig. 1F). Many published papers on different cancer types have focused their investigative efforts on downregulated genes upon BETi administration. However, the genes induced after JQ1 treatment could provide an unexplored opportunity to discover drug-induced vulnerabilities in TNBC cells. The first GO (Gene Ontology) category of genes upregulated upon JQ1 treatment belongs to the metabolic pathway, suggesting BET proteins’ role in metabolic rewiring through chromatin regulatory mechanisms. Among the 127 genes of the metabolic pathway category, 25% belong to the lipid metabolic process (Fig. 1G). Since the role of BET proteins on lipid metabolism is poorly characterized, we decided further to characterize these genes (Fig. 1H). We confirmed by RT-qPCR that JQ1 leads to the upregulation of several lipid metabolism genes (ACER2, PNPLA2, DGAT1, SMPD3, SYNJ1 and MTM1) in both our TNBC cell line models, MDA-MB231 and Hs578t (Fig. 1I and Supplementary Fig. 1B).

### BET inhibitors alter metabolic reprogramming lowering intracellular lipid droplets and fatty acids content

**Fig. 2:**
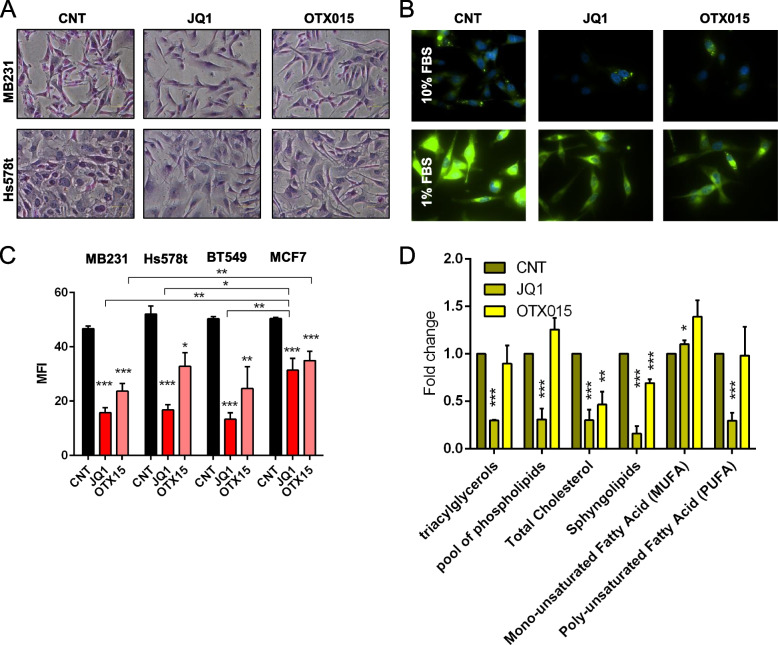
BETi provoke metabolic reprogramming lowering intracellular LDs content. **A** Representative images of MDA-MB231 and Hs578t cells stained with ORO staining after 24 hours of 1 μM BETi treatment. **B** Representative images of MDA-MB231 cells stained with BODIPY 500/510 growth in normal condition (10% FBS, above) and in overnight starvation (1% FBS, below) after 24 hours of 1 μM BETi treatment. **C** Flow cytometry analysis of LDs content stained with BODIPY 500/510 in BC cell lines after 24 hours of 1 μM BETi treatment (MFI = median fluorescence intensity). **D** NMR analysis of neutral lipid content in MDA-MB231 cells after 24 hours of 1 μM BETi treatment

We investigated whether the increased expression of lipid metabolism genes upon JQ1 treatment reflected a phenotypic change in TNBC cell lines. Oil red O (ORO) staining after 24 hours of JQ1 and OTX015 treatment, revealed a considerable decrease of lipid droplets (LDs) in both TNBC cell lines (Fig. 2A). These results were confirmed with a fluorescent fatty acid analog (BODIPY 500/510) staining used as a live cell marker of LDs [28]. BODIPY 500/510 signal was decreased after BETi treatment (Fig. 2B, top). Because TNBC cells appear to be more dependent on exogenous FAs uptake and storage, we investigated whether BETi had a similar effect on LDs biogenesis under deprived growth conditions (1% FBS overnight). Low serum settings represents the most robust induction model of LDs formation [29]. Likewise, BETi also reduced LDs content in nutrient deprivation conditions (Fig. 2B, bottom). We also quantified the LDs using the BODIPY 500/510 dye in flow cytometry after 24 hours of BETi treatment. All TNBC treated cells displayed a significant decrease in fluorescence intensity compared to MCF7 treated cells (Fig. 2C). On MDA-MB231 and Hs578t cells, LDs reduction was already evident after 6 hours of treatment (Supplementary Fig. 1C). By proton NMR spectra profile, we identified and quantified different classes of neutral lipids, such as triglycerides, phospholipids, cholesterol, sphingolipids, and mono-unsaturated fatty acids (MUFAs), and poly-unsaturated fatty acids (PUFAs). Again, in MDA-MB231, we detected the most substantial decrease in neutral lipid, the main constituents of LDs, upon JQ1 treatment compared to OTX015 (Fig. 2D). To a lesser extent, we observed a decrease in TAG in Hs578T cells (Supplementary Fig. 2A). Although BET inhibition significantly lowers LDs content in TNBC cells, the lipid composition in the Hs578T cell line, upon BETi treatment, is affected differently than the MDA-MB231. An explanation for these results may be due to a shift to other structural lipids not detectable in these settings by NMR.

### BETi promote LDs lipolysis by increasing PNPLA2/ATGL expression and function

**Fig. 3:**
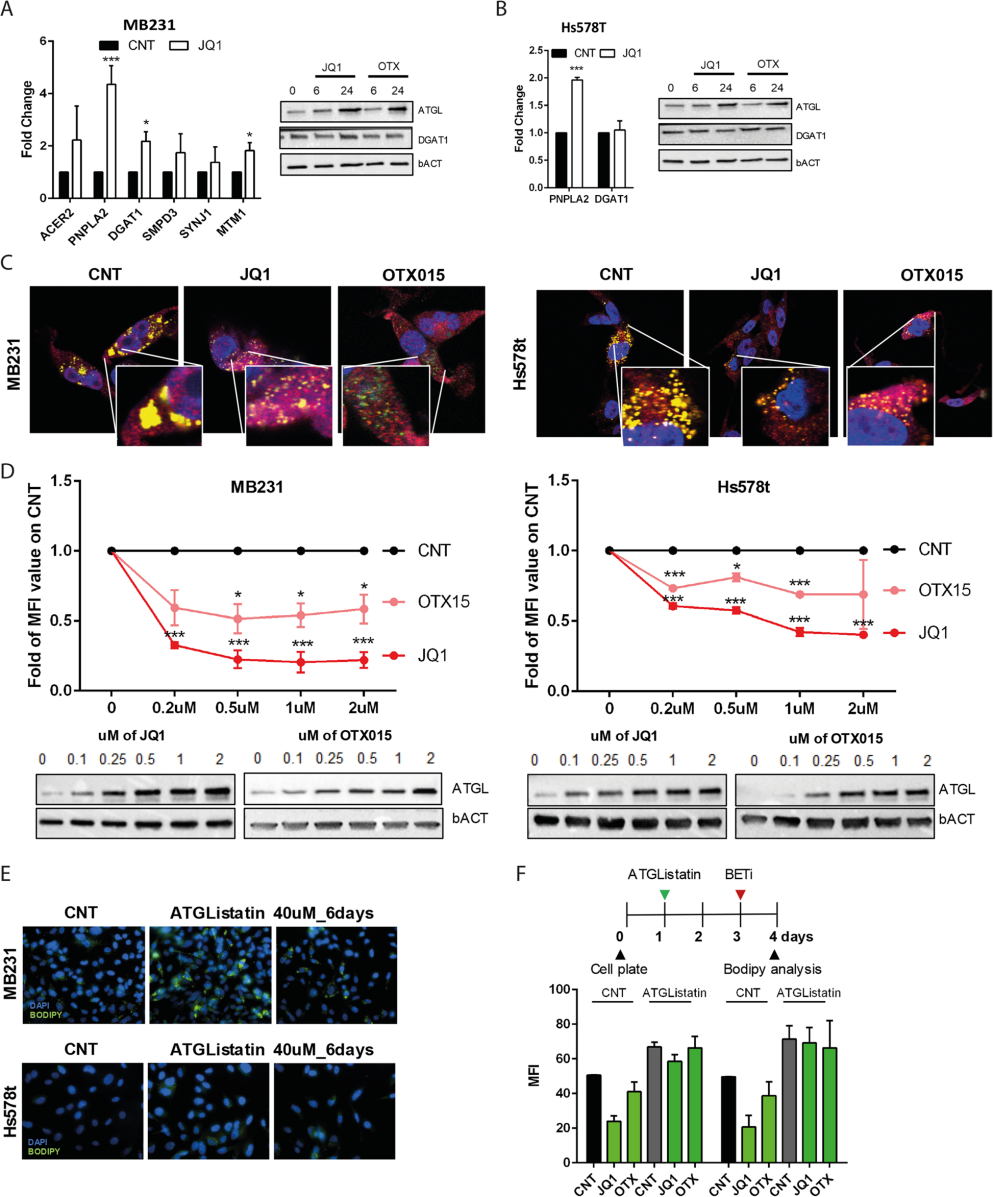
PNPLA2/ATGL is responsible for LDs mobilization upon BETi treatment. **A, B** Gene and protein expression in MDA-MB231 and Hs578t after 1 μM BETi treatment for the indicated time. **A** Left, RT-qPCR expression analysis on lipid metabolic genes selected in Fig. 1I after 24 hours of BETi treatment. Right, western blot analysis showed ATGL and DGAT1 protein expression in MDA-MB231. **B** Left, RT-qPCR results on PNPLA2 and DGAT1 genes in Hs578t after 24 hours of BETi treatment. Right, western blot analysis showed ATGL and DGAT1 protein expression in Hs578t. **C** Representative immunofluorescence images on MDA-MB231 and Hs578t treated with 1 μM of BETi for 24 hours. Green is BODIPY 500/510; red is ATGL, and blue is DAPI (nuclei staining). **D** Flow cytometry analysis of LDs content stained with BODIPY 500/510 in MDA-MB231 and Hs578t after 24 hours of BETi treatment at different concentrations (from 0.2 to 2 μM) and, below, ATGL expression analysis in both cell lines after 24 hours of BETi treatment from 0.1 to 2 μM. **E** Representative immunofluorescence images of MDA-MB231 and Hs578t cells after 6 days of treatment with 40 μM ATGListatin (green indicates LDs stained with BODIPY 500/510). **F** On top, a schematic timeline of the treatment. Below, a flow cytometry analysis of LDs content stained with BODIPY 500/510 in MDA-MB231 and Hs578t cells treated with ATGListatin and BETi. bACT is used as a loading control in both cell lines

Among the six genes significantly upregulated after 6 hours of JQ1 treatment (Fig. 1I), PNPLA2, which encodes for the adipose triglyceride lipase-ATGL, was the most upregulated in both MDA-MB231 and Hs578t-treated cells after 24 hours of BETi treatment (Fig. 3A and B). ATGL localizes on the LDs surface and is universally recognized as the critical enzyme of LDs lipolysis in both adipose and non-adipose tissues [13–15]. ATGL activity is counterbalanced by another enzyme, DGAT1, primarily involved in forming new LDs [30, 31]. Upregulation of ATGL protein and the modest changes in DGAT1expression level upon BETi treatment in MDA-MB231 (Fig. 3A) and Hs578t (Fig. 3B) cells led us to hypothesize that LDs mobilization is linked to an increase in lipolysis driven by ATGL rather than a deficiency in LDs formation. LDs lipolysis by ATGL after BETi treatment was independently confirmed by confocal microscopy, where a reduction in the size and localization of LDs (BODIPY staining, green) is evident and is sustained by a disperse localization of the endogenous ATGL signals (Fig. 3C, red). We confirmed the overexpression of ATGL in another TNBC cell line, BT549 cells, while MCF7 cells only slightly overexpressed ATGL after 24 hours of BETi treatment (Supplementary Fig. 2B). We then assessed a correlation between ATGL and LDs mobilization. BETi decreased LDs signal according to with ATGL induction in a dose-dependent manner in both cell lines (Fig. 3D). We confirmed ATGL’s role in regulating LDs mobilization treating TNBC cell lines with a pharmacological inhibitor of ATGL function, ATGListatin. ATGListatin treatment induced LDs accumulation in MDA-MB231 and Hs578t cells (Fig. 3E). To confirm that ATGL function is needed for BETi-induced lipolysis to exert their antitumoral effects, we pre-treated MDA-MB231 and Hs578t cells with control (CNT) and ATGListatin for 3 days before adding BETi for the last 24 hours to both conditions. CNT-treated cells displayed a reduction of the BODIPY signal after BETi treatment, while ATGListatin-treated cells showed any significant changes (Fig. 3F). Taken together, these results showed that ATGL plays a key role in regulating the lipid network of TNBC cells induced by BETi.

### BETi affect cell proliferation in FAs dose-dependent manner helped by ATGL mediating lipolysis

**Fig. 4:**
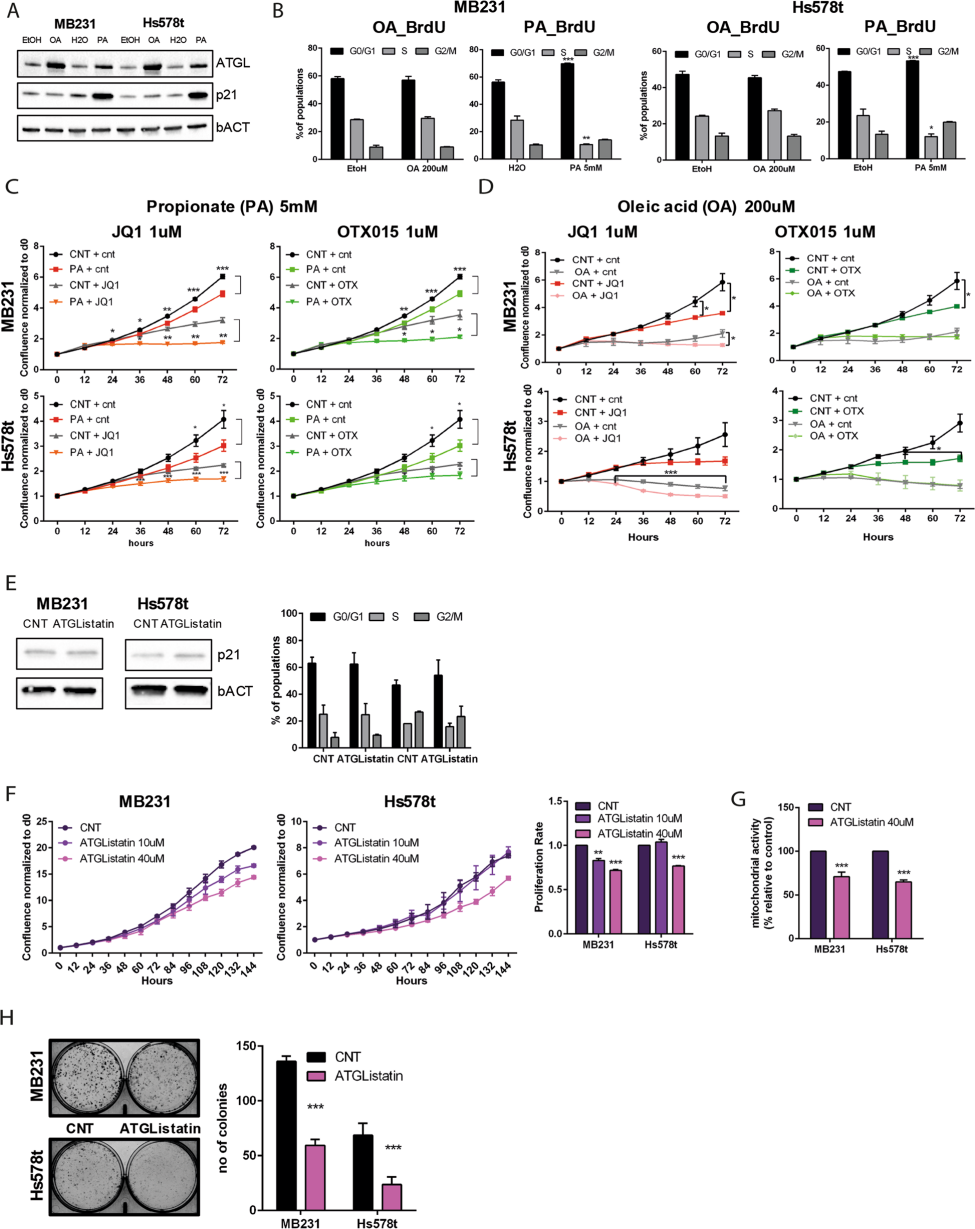
Influence of fatty acids (FAs) on cell proliferation. **A** Immunoblotting for ATGL and p21 in MDA-MB231 and Hs578t cells after treatment with 200uM of Oleic Acid (OA) and 5 mM of Propionic Acid (PA) with the respective vehicle (EtOH and H2O respectively) for 24 hours. **B** Cell cycle analysis of MDA-MB231 and Hs578t cells treated with OA and PA for 24 hours after 1 hour pulse of BrdU. BrdU and PI incorporation indicated the % of the population. Histograms are the % of cell population in all three different phases of the cell cycle. The significance is calculated versus the CNT treated cells. **C, D** Cell proliferation assay measured by IncuCyte to follow the effect of PA 5 mM (**C**) and OA 200uM (**D**) in MDA-MB231 and Hs578t in combination with 1 μM BETi treatment. **E** Left, immunoblotting for p21 in MDA-MB231 and Hs578t cells after ATGListatin treatment for 3 days. Right, cell cycle analysis of MDA-MB231 and Hs578t cells treated with ATGListatin for 3 days followed 1 hour pulse of BrdU. Histograms are the % of cell population in all three different phases of the cell cycle. **F** Left, cell proliferation assay measured by IncuCyte and, right, endpoint (6 days) to evaluate the effect of different ATGListatin concentrations in MDA-MB231 and Hs578t. **G** MTT assay to assess ATGListatin on mitochondrial metabolic activity after 6d of treatment at 40 μM. **H** Left, representative images of colony formation assay (cristal violet dye) in MDA-MB231 and Hs578t cells treated with ATGListatin 40 μM and right, quantification of the colonies by ImageJ sofware. bACT served as a loading control

Although FAs are considered necessary energy sources, when their intra- and/or extracellular concentrations exceed the physiological levels they become lipotoxic inducing mitochondria oxidative stress, and cell death. To prevent this fate, cancer cells exploit the DGAT1 function to channel the FAs into LDs. In TNBC cells, BETi treatment did not affect DGAT1 expression (Fig. 3A and B). Therefore, we hypothesize that TNBC cells might not be able to tolerate an excess of free FAs due to ATGL overexpression. To prove our theory, we administrated oleic acid (OA) and propionic acid (PA) to MDA-MB231 and Hs578t cells. OA is one of the most abundant FAs in the cells and a potent inducer of LDs accumulation and PA is a short-chain FA that easily crosses the mitochondrial membrane mimicking ATGL activity. Although 100uM of OA and 0.5/1 mM of PA added to MDA-MB231 and Hs578t are still in the physiological limit tolerance of the cells, increased doses of both FAs affected cell proliferation (Supplementary Fig. 2C). Interestingly, OA and PA affected ATGL expression and cell cycle progression differently. PA treatment (5 mM) for 24 hours showed results similar to the BETi, with a strong upregulation of p21 expression and G0/G1-phase arrest in both MDA-MB231 and Hs578t cells (Fig. 4A-B). Instead, 24 hours of OA (200uM) did not alter p21 expression and cell cycle progression, but drastically induced ATGL expression (Fig. 4A-B). As expected, in both cell lines, we achieved a synergistic effect on cell growth reduction when PA and BETi were used together (Fig. 4C). This effect is less appreciable when OA is added to BETi (Fig. 4D). These data suggest that the mitochondria, overfed by an excess of FAs, impaired the capability of the MDA-MB231 and Hs578t cells to grow. To confirm that ATGL mediating lipolysis helped BETi to affect cell proliferation, we inhibited ATGL function with the selective inhibitor ATGListatin. After 3 days of ATGListatin administration on MDA-MB21 and Hs578t cells, we did not detect changes in p21 expression (Fig. 4E, left), and the cell cycle phases (Fig. 4E, right). In fact, functional inhibition of ATGL reduced proliferation (Fig. 4F), mitochondrial metabolic activity (Fig. 4G), and the ability to colony-forming of TNBC cells (Fig. 4H). This cellular phenotype is not driven by alteration of the cell death (Supplementary Fig. 2E), but it could be attributed to the reduced availability of FAs and energy supply. Together, these data demonstrated that BETi reduced the LDs involves an upregulation of the ATGL function that synergistically impacts on the growth arrest.

### The downregulation of ATGL improves mitochondrial function and promotes a metabolic switch in TNBC cells

**Fig. 5:**
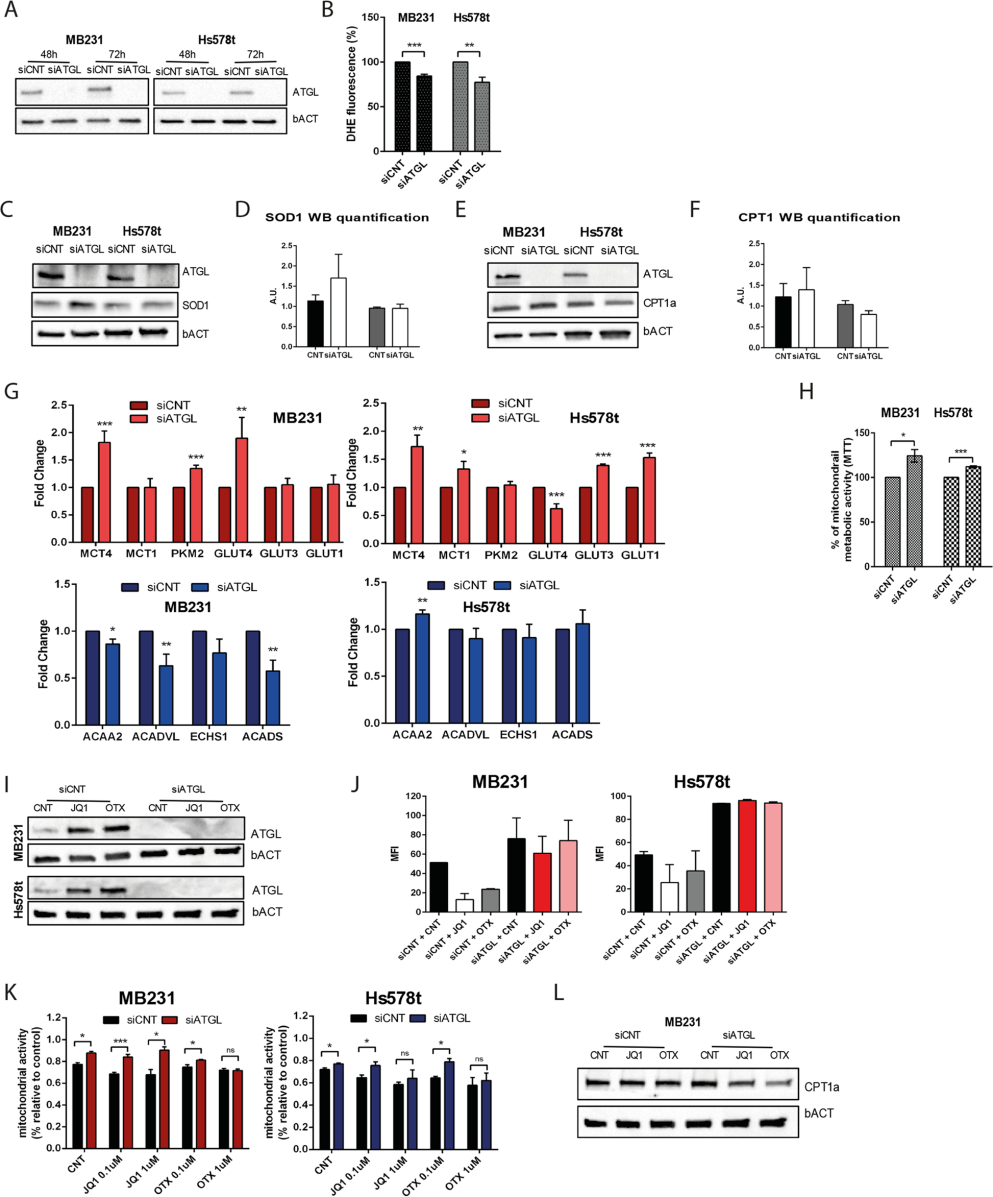
ATGL downregulation influences mitochondrial and metabolic dynamics in TNBC cells. **A** Western blot analysis showing ATGL downregulation in MDA-MB231 and Hs578t in control (siCNT) and ATGL siRNA cells (siATGL) after 48 and 72 hours from siRNA transfection. **B** DHE fluorescence measure to evaluate the ROS content after 48 hours of ATGL downregulation. **C**, **D** Protein expression analysis of SOD1 and ATGL (**C**) and (**D**) SOD1 band quantification in MDA-MB231 and Hs578t cells after 48 h of ATGL downregulation. **E-F** Protein expression analysis of CPT1a and ATGL (**E**) and (**F**) CPT1a band quantification in MDA-MB231 and Hs578t cells after 48 h of ATGL downregulation. **G** RT-qPCR analysis to evaluate the effect of ATGL downregulation (siRNA 48 h) on glycolytic (upper panel) and β-oxidation (bottom panel) genes in MDA-MB231 and Hs578t. **H** MTT assay to evaluate the mitochondrial metabolic activity in MDA-MB231 and Hs578t after 48 h of siATGL transfection. **I-L** Schedule: 48 h of siRNA transfection (siCNT and siATGL) and the last 24 h 1 μM of BETi treatment in MDA-MB231 and Hs578t. **I** ATGL expression analysis and (**J**) flow cytometry analysis of LDs content stained with BODIPY 500/510. **K** MTT assay with two different doses (0.1 and 1 μM) of BETi. **L** CPT1a protein expression analysis. Representative images are selected for the western blot. bACT served as a loading control

To better understand the role that ATGL plays in mediating BETi-induced LDs lipolysis, we interfered with ATGL in both TNBC cell lines (Fig. 5A). Similarly, to the ATGListatin results, siATGL decreased proliferation and increased in LDs content, and did not change in p21 expression (Supplementary Fig. 3A-C). Previous publications showed that JQ1 treatment changes mitochondrial dynamics, inducing mitochondrial dysfunction and increasing oxidative stress [32–34]. We found that ATGL downregulation ameliorated the harmful effects of oxidative stress induced by ROS (Reactive Oxygen Species), decreasing their accumulation in both TNBC cell lines (Fig. 5B). Interestingly, MDA-MB231 cells decreased ROS upregulating Superoxide Dismutase 1 (SOD1), a major antioxidant enzyme (Fig. 5C-D) while Hs578t cells decreased the oxidative stress downregulating Carnitine Palmitoyltransferase 1a (CPT1a), the enzyme that controls the mitochondrial FAs uptake and determines the ß-oxidation flux (Fig. 5E-F). Given the pivotal role of ATGL in lipid metabolism and glycolysis as a strategy for rapid ATP synthesis with less ROS in cancer cells [35], a possible contribution of ATGL downregulation to the switch from mitochondrial metabolism to a glycolytic phenotype was investigated. We observed an increased expression of several glycolytic markers (Fig. 5G, up) and decreased ß-oxidation genes (Fig. 5G, bottom), indicating a rewired metabolism toward a glycolytic asset. The enhanced glycolysis reflected an improved mitochondrial metabolic activity measured by MTT assay in siATGL compared with the siCNT (Fig. 5H). Taking these data altogether, we conclude that ATGL activity is required for mitochondrial lipid catabolism. However, MDA-MB231 and Hs578t cells acquired a hybrid metabolic state by demand-sensitive crosstalk of regulatory proteins and energy, mirroring the dissimilarity of their metabolic rewiring upon ATGL interference. Furthermore, to confirm that ATGL-mediated lipolysis helped the antiproliferative effect of BETi bursting under forced lipid catabolism, we interfered with ATGL and treated both cell lines with BETi (Fig. 5I). Again, the BETi effects on the LDs mobilization were entirely abolished in the absence of ATGL (Fig. 5J), as their metabolic activity is less affected by the treatment (Fig. 5K). Intriguingly, CPT1a levels were lower in MDA-MB231 and Hs578t cells siATGL treated with BETi, corroborating our hypothesis that BETi needs ATGL to exert their antiproliferative effects through enhanced mitochondrial lipid catabolism (Fig. 5L and Supplementary Fig. 3D). Together, these data demonstrated that mitochondrial dysfunction/oxidative stress induced by BETi also leaned on the lipase activity of ATGL.

### FoxO1 modulates ATGL expression

**Fig. 6:**
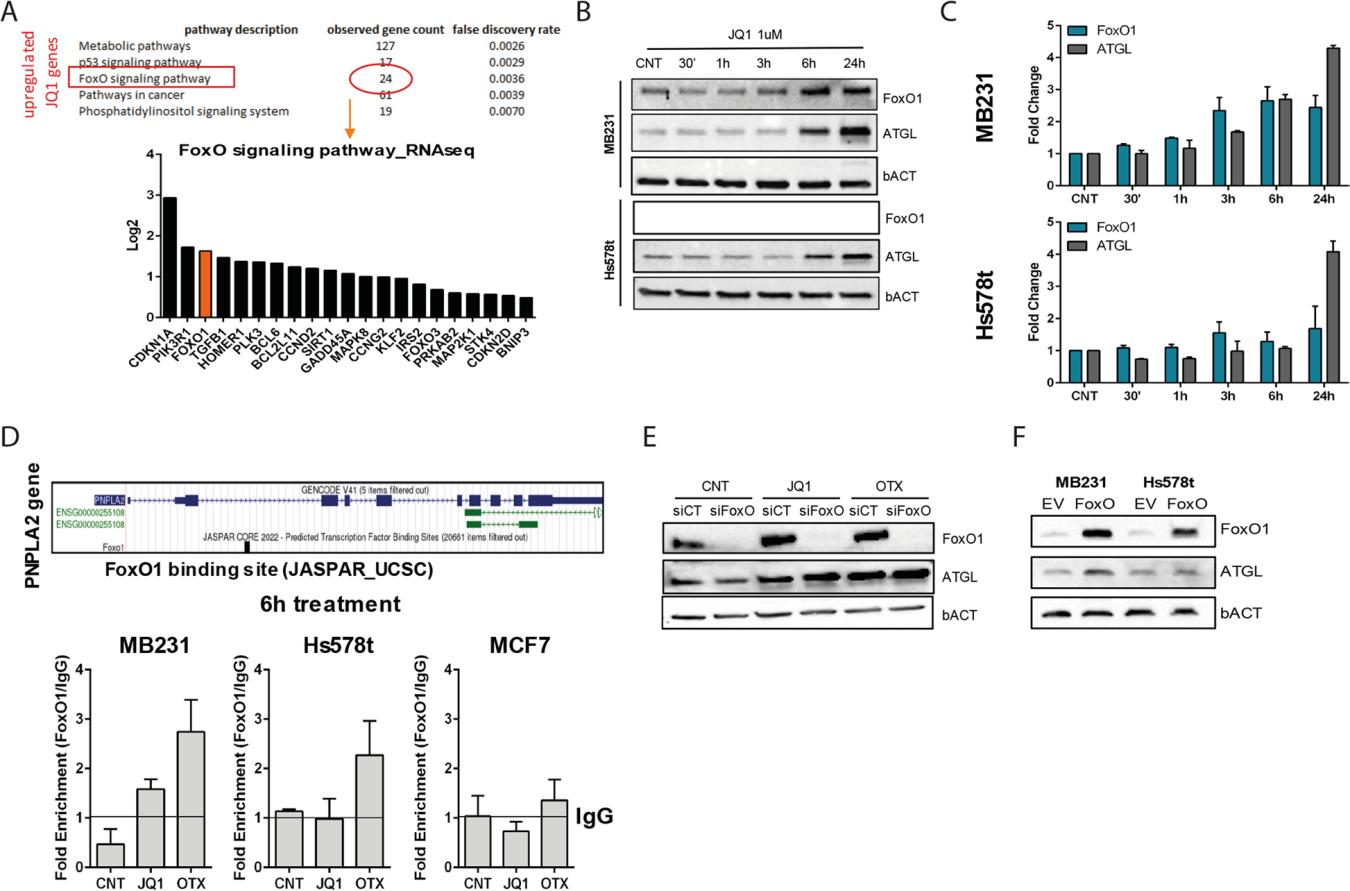
BETi cooperate with FoxO1 to mediate ATGL transcriptional activation. **A** The top 5 GO (gene ontology) categories as in Fig. 1G and the plot of the log2 fold change value of FoxO signaling pathway category; red indicates FoxO1. **B** Time-course (30 minutes to 24 hours) protein expression analysis of FoxO1 and ATGL in MDA-MB231 and Hs578t after 1 μM JQ1 treatment. **C** RT-qPCR analysis of FoxO1 and ATGL (PNPLA2 gene) after 1 μM JQ1 treatment at different time points (30 minutes to 24 hours). **D** ChIP analysis of FoxO1 binding on PNPLA2 promoter after 6 hours of 1 μM BETi treatment in MDA-MB231, Hs578t and MCF7 cell lines. The TAAACT FoxO1 binding site is located around 1.6Kb downstream of the TSS. The line indicates the IgG value **E** Protein expression analysis of ATGL and FoxO1 in control (siCNT) and FoxO1 siRNA cells (siFoxO) after 48 hours of siRNA transfection combined with 1 μM BETi for the last 24 hours. **F** Western blot analysis of FoxO1 and ATGL in MDA-MB231 and Hs578t in control (EV) and FoxO1 overexpression (FoxO) after 24 hours from transfection. Representative images are selected for the western blot. bACT served as a loading control

RNA-seq data on MDA-MB231 cells following JQ1 treatment revealed the upregulation of several genes belonging to the forkhead homeobox (FoxO) signaling pathway (Fig. 6A and Supplementary Fig. 4A). Among those genes, we found FoxO1, a transcription factor member of the FoxO family, which can stimulate ATGL expression [36]. Moreover, FoxO1 inhibits G0/G1 gene 2 (G0S2), an inhibitor of ATGL expression [37]. To investigate if FoxO1 is implicated in BETi-stimulated ATGL expression, we initially evaluated the effect of BETi on the expression of FoxO1 and ATGL over time. MDA-MB231 and Hs578t cells with BETi increased FoxO1 mRNA already after 3 hours, and FoxO1 and ATGL protein after 6 hours (Fig. 6B-C and Supplementary Fig. 4B). FoxO1 is upregulated also in BT549 cells but not in MCF7 cells after BETi treatment (Supplementary Fig. 4C). To determine whether BETi not only improved protein expression but may induce transcriptional activation thought a direct bonding of FOxO1 to the PNPLA2 promoter, we performed a ChIP assay. The PNPLA2 genome region presents a FoxO1 binding site 1.6 kb downstream of the TSS, predicted on JASPAR at the UCSC browser. The ChIP assay on MDA-MB231, HS578t and MCF7 treated for 6 h with BETi showed the binding of FoxO1 to the PNPLA2 promoter only after the treatment in TNBC cells (Fig. 6D). Next, we sought to determine whether ATGL transcriptional activation may depended on FoxO1 after BETi administration. In cells treated for siFoxO1 compared to CNT, we observed a reduced the expression of ATGL. However, the effect was rescued by BETi treatment (Fig. 6E). Therefore, induction of ATGL expression by BETi is not completely dependent on changes in the endogenous FoxO1 expression, suggesting that other mechanisms play a role in its activation. On the contrary, the overexpression of FoxO1 [38] induced the expression of ATGL, confirming FoxO1 involvement in ATGL regulation (Fig. 6F). We also rated G0S2 expression as a selective regulator of ATGL. The G0S2 mRNA expression [39] is significantly downregulated after BETi treatment, leading to the increased lipase activity of ATGL (Supplementary Fig. 4D). Together, these results indicate that BETi treatment induces ATGL expression in collaboration, but not directly mediated, with FoxO1.

## Discussion

Triple-negative breast cancer (TNBC) is a highly aggressive subtype of breast tumor that accounts for 10–15% of all breast cancer (BC). The high mortality of this tumor is due to the lack of therapeutic options. If TNBC relapses, evades therapy and/or develops distant metastasis, patients undergo to a reduced life expectancy. Lipid metabolism, and metabolism in general, is a fundamental process to assure the proper development of cellular functions, but also sustains tumor proliferation and aggressiveness (reviewed in [40, 41]), when lipid balances are altered. However, the cellular and molecular mechanisms that regulate lipid metabolism alterations are not yet completely elucidated. In this study, we employed a class of epigenetic drugs, BET inhibitors (BETi), used in vitro and in clinical trials for different malignancies, to unveil BET protein’s role in controlling the lipid metabolism in TNBC cells. First, BET proteins can directly or indirectly influence the expression of genes belonging to metabolic pathways, in particular the ones involved in lipid metabolism. Although the involvement of BET proteins in the regulation of lipid homeostasis is still elusive, our results demonstrated that BET proteins inhibition induced an overall reduction of the neutral lipid stored in the cytosolic LDs. BETi promoted the ATGL-mediated TAG hydrolysis to release FAs from LDs that overfeed the mitochondria inducing p21 upregulation and inducing growth arrest.

In particular, the induction of ATGL function disrupts the lipid homeostasis with an excessive free FAs shuttling into the mitochondria, which leads to an increase of the oxidative stress that impacts the proliferative capability of TNBC cells. Our data showed that the inhibition of ATGL function resulted in the reduction of oxidative stress differently in MDA-MB231 and Hs578t cells. Hs578t cells downregulated CPT1a protein and its reduction can facilitate the entry of excess FAs into mitochondria for β-oxidation, leading to a remarkable drop in ROS production. MDA-MB231 cells increased SOD1 protein expression, which is responsible for cell detoxification. Second, BET proteins also impact mitochondrial dynamics and functions. In absence of ATGL, the effect of BETi on LDs mobilization is abolished and, together with the reduced CPT1a expression, attenuated the strong effect that BETi have on mitochondrial vitality in both MDA-MB231 and Hs578t cells. Interestingly, these molecular effects are also coherent with the ability of tumor cells to switch from an oxidative to a glycolytic status to obtain faster glucose-derived energy in absence of ATGL. As confirmation of this switch, we detected an upregulation of genes involved in glycolysis and a decreased expression of genes associated with β-oxidation. Our data indicated that silencing of ATGL reduced TNBC cell proliferation, confirming the dependency of TNBC cells on FAs and lipids as a source of energy to proliferate. The lack of FAs availability, paralleled to a metabolic rewiring, allows cells to avoid cell death, as we detected in our study. Although the function of ATGL is unique, the molecular outcomes on MDA-MB231 and Hs578t cells are different. An elevated heterogeneity characterizes TNBC. As well as TNBC cells, the heterogeneity follows the in vivo complexity. Therefore, it is expected to have a discrepancy in the behavior of these two cell lines. Moreover, different studies attribute a controversial role of ATGL in the regulation of tumorigenesis in a variety of human malignancies [42–46]. At the same time, ATGL upregulation in BC is associated with an enriched adipocyte tumor microenvironment (TME), contributing to the aggressiveness of high-grade tumors [47]. Thus, the role of ATGL remains elusive in these TNBC tumors. Hence, a better understanding of these processes could pave the way for the development of therapies targeting lipid metabolism, providing new strategies to eradicate the whole tumor mass in cancers with unmet needs and high degree of heterogeneity. Here, we can conclude that tumors might take advantage of ATGL deregulation to suppress the non-energetic functions, which would otherwise hinder tumor promotion/progression. In our experimental setting, the role of ATGL might be critical to BETi to exert their antiproliferative effect, whether ATGL downregulation might contribute to the switch from mitochondrial metabolism to a glycolytic phenotype typical of many cancers. This led us to speculate a divergent role for ATGL under the stressful condition driven by BETi. Finally, FoxO1 and its pathway are upregulated in the RNA-seq performed after JQ1 treatment in the MDA-MB231 cell line. FoxO1, an upstream activator of ATGL, is bound to the PNPLA2 promoter only after BETi administration. However, this partially elucidate the mechanisms of BETi-modulating abnormal lipolysis in TNBC: increased expression of FoxO1 caused the upregulation of ATGL, but it contributed only partially to this upregulation. In fact, in FoxO1 silenced cells BETi rescued ATGL expression. It is reasonable to think that the regulation of ATGL might depend on different pathways. Nowadays, there are many studies on the role of ATGL in cancer, but there is a lack of studies on transcriptional factors that target the PNPLA2 promoter in tumor cells. Silencing of BRD4 affected ATGL mRNA expression after 72 hours (data not shown). However, further experiments are needed to assign a direct role of BRD4 in the modulation of LDs content. Additional experiments are also needed to unveil the molecular mechanisms that lead to the activation of ATGL upon BETi administration.

## Conclusions

Our research defined that, among the plethora of pathways dysregulated by BETi, the lipolysis mediated by ATGL could be fundamental to help BETi to reduce tumor proliferation and growth. Furthermore, ATGL might also be the link between BETi treatment and the changes in mitochondrial dynamics derived by the usage of these inhibitors. When high levels of exogenous FAs are present, BETi have a synergic effect on growth arrest, opening a new approach to TNBC therapy in obese patients. FAs are an integral part of lipid metabolism, and FAs’ role in obesity-related morbidity is of great interest as obesity is a chronic disease that promotes the progression and metastasis of breast cancer. Noteworthy, this mechanism seems to be peculiar to TNBC cells.

## Supplementary Information

**Additional file 1: Supplementary Fig. 1. A.** CFSE analysis of MDA-MB231 and HS578t after 24–48-72 hours of 1uM JQ1 treatment. Fluorescence decay is relative to the fluorescence at 24 hours of treatment **B.** RT-qPCR analysis of RNA-seq selected genes validation in Hs578t after 6 h treatment with JQ1 1 μM. **C.** Left, representative flow cytometry curves of BODIPY 500/510 fluorescence signal in MDA-MB 231 and Hs578t after 24 hours of 1 μM BETi treatment Right, flow cytometry analysis of LDs content stained with BODIPY 500/510 in MDA-MB 231 and Hs578t cell lines after 6 and 24 h of 1 μM BETi treatment (MFI = median fluorescence intensity). **Supplementary Fig. 2. A.** NMR analysis of metabolite content in Hs578t after 24 hours of 1 μM BETi treatment. **B.** Protein expression analysis of ATGL in all BC cell lines for 24 hours with 1 μM of BETi**. C.** Cell proliferation assay of MDA-MB231 and Hs578t measured by IncuCyte after adding different concentrations of FAs. OA: Oleic Acid; PA: Propionic Acid. **D.** CFSE analysis of MDA-MB231 and HS578t after 24–48-72 hours of 100uM and 200uM of oleic acid (OA, left) or 1 mM and 5 mM of propionate (PA, right) treatment. Fluorescence decay is relative to the fluorescence at 24 hours of treatment**. E.** Percentage of cell death in MDA-MB231 and Hs578t cells treated with 3 days with ATGListatin and stained with Annexin V. Representative images are selected for western blot. bACT served as a loading control. **Supplementary Fig. 3.** A. Cell proliferation assay of MDA-MB231 and Hs578t measured by IncuCyte after silencing ATGL. **B.** Flow cytometry evaluation of LDs content stained with BODIPY 500/510 in MDA-MB231 and Hs578t after 48 and 72 hours of siRNA transfection (siCNT vs siATGL). **C.** Immunoblotting for ATGL and p21 in MDA-MB231 and Hs578t cells after 48 hours of siRNA transfection (siCNT vs siATGL). **D.** Protein expression analysis of CPT1a after a combination of siRNA approach (siATGL for 48 h) and BETi treatment (1 μM for the last 24 hours) in Hs578t. Representative images are selected for the western blot. bACT served as a loading control. **Supplementary Fig. 4. A.** Validation of RNA-seq analysis of MDA-MB231 treated for 6 hours with 1 μM of JQ1 (FoxO signaling pathway). **B.** Time-course (30 minutes to 24 hours) protein expression analysis of ATGL and FoxO1 in MDA-MB231 and Hs578t after 1 μM OTX-015 treatment. **C**. Protein expression analysis of FoxO1 in BT549 and MCF7 cell lines after 6 hours of 1 μM BETi. **D.** RT-qPCR analysis of FoxO1 and G0G2 in MDA-MB231 and Hs578t after 24 hours treatment with 1 μM of BETi. Representative images are selected for the western blot. bACT served as a loading control.

## Data Availability

All data generated and/or analyzed during the current study are available from the corresponding author on reasonable request.

## Abbreviations

ACAA2: Acetyl-CoA Acyltransferase 2
ACADS: Acyl-CoA Dehydrogenase Short Chain
ACADVL: Acyl-CoA Dehydrogenase Very Long Chain
ACER2: Alkaline Ceramidase 2
ATGL: Adipose Triglyceride Lipase
BC: Breast Cancer
BET: Bromodomain and Extraterminal domain
BETi: BET inhibitor
BrdU: 5-bromo-2′-deoxyruridine
CFSE: 5(6)-Carboxyfluorescein N-hydroxysuccinimidylester
ChIP: Chromatin Immunoprecipitation
CPT1a: Carnitine Palmitoyltransferase 1a
DGAT1: Diacylglycerol O-Acyltransferase 1
DHE: Dihydroethidium
ECHS1: Enoyl-CoA Hydratase, Short Chain 1
FA(s): Fatty Acid(s)
FAO: Fatty Acid Oxydation
FITC: Fluorescein isothiocyanate
GLUT1: SLC2A1 Solute Carrier Family 2 Member 1
GLUT3: SLC2A3 Solute Carrier Family 2 Member 3
GLUT4: SLC2A4 Solute Carrier Family 2 Member 4
GO: Gene Ontology
H: Hour(s)
IF: Immunofluorescence
KD: Knockdown
LD(s): Lipid Droplet(s)
MCT1: SLC16A1 Solute Carrier Family 16 Member 1
MCT4: SLC16A4 Solute Carrier Family 16 Member 4
MFI: Median Fluorescence Intensity
MTM1: Myotubularin 1
MTT: 1-(4,5-Dimethylthiazol-2-yl)-3,5-diphenylformazan
MUFA: Mono-Unsaturated Fatty Acid
NMR: Nuclear Magnetic Resonance
OA: Oleic Acid
ORO: Oil Red O
PA: Propionic Acid
PFA: Paraformaldehyde
PI: Propidium Iodide
PKM2: Pyruvate Kinase M1/2
PLB: Passive Lysis Buffer
PNPLA2: Patatin Like Phospholipase Domain Containing 2
PUFA: Poly-Unsaturated Fatty Acid
RNA-seq: RNA-sequencing
ROS: Reactive Oxygen Species
RT-qPCR: Reverse-transcription quantitative PCR
siRNA: small interfering RNA
SMPD3: Sphingomyelin Phosphodiesterase 3
SOD1: Superoxide Dismutase 1
SYNJ1: Synaptojanin 1
TAG: triacylglycerol
TMS: Tetramethylsilane
TNBC: Trple-Negative Breast Cancer
TSP: 3-(trimethylsilyl) propionic-2,2,3,3-d4 acid sodium salt
