# Supplementary figures and images for: BETi enhance ATGL expression and its lipase activity to exert their antitumoral effects in triple-negative breast cancer (TNBC) cells
Source: J Exp Clin Cancer Res. 2023 Jan 6;42:7. doi: 10.1186/s13046-022-02571-3 (PMC9817244; doi:10.1186/s13046-022-02571-3)

Supplementary figure 1

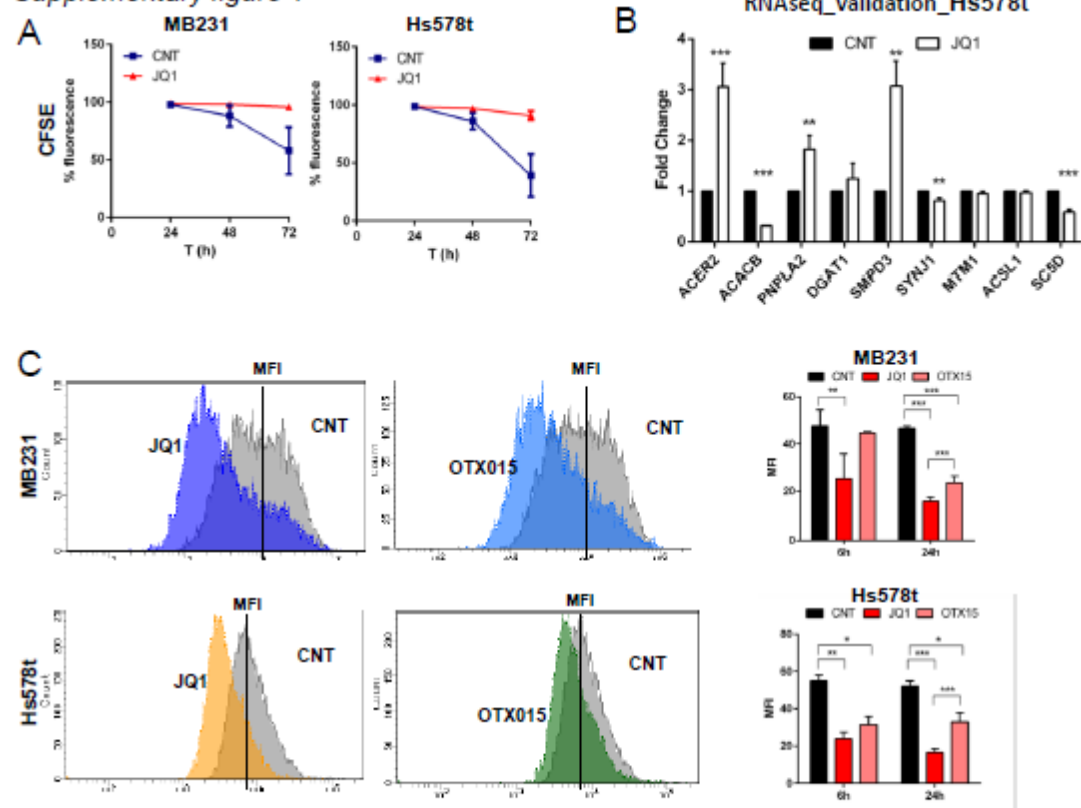

Supplementary figure 2

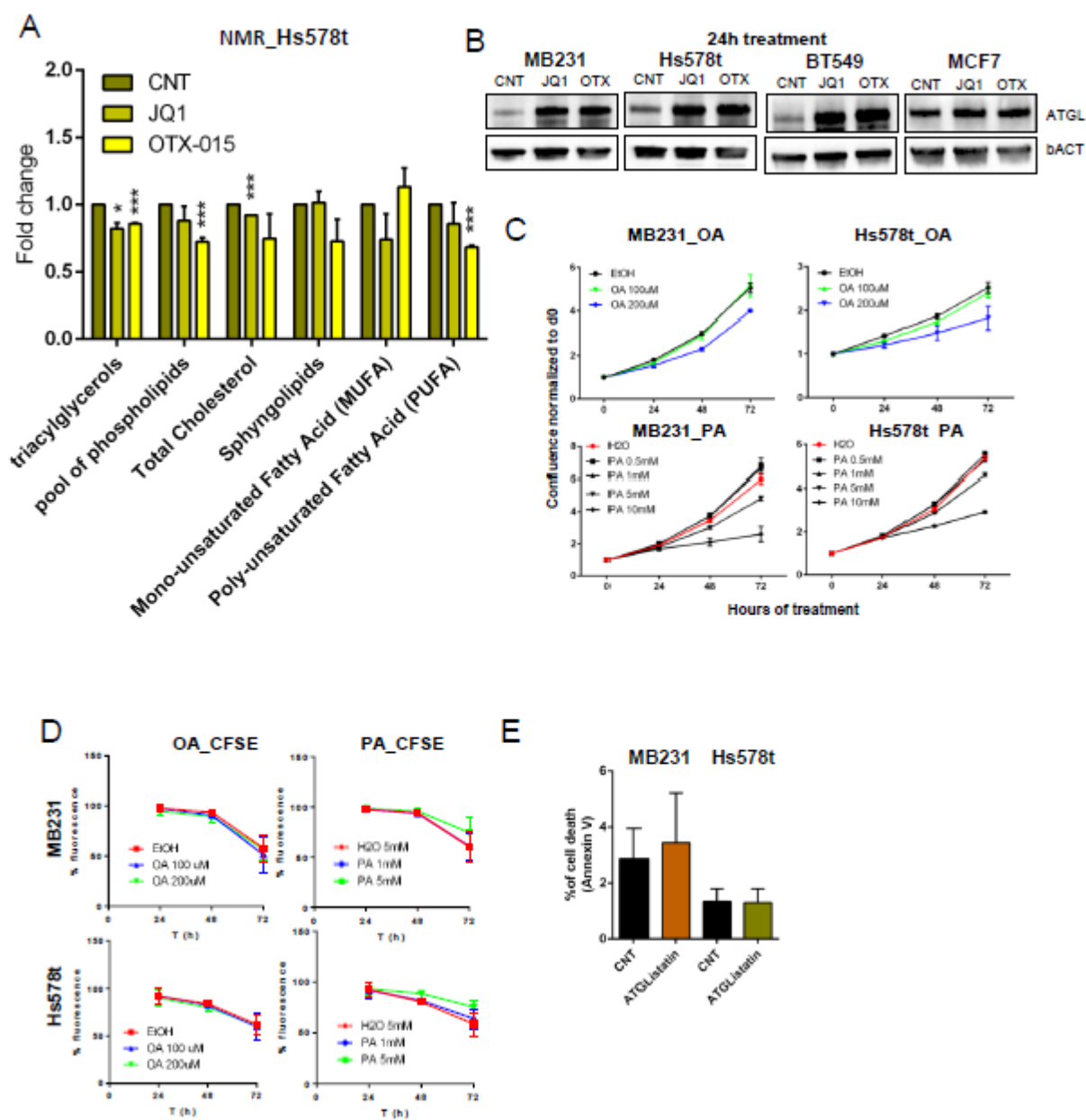

Supplementary figure 3

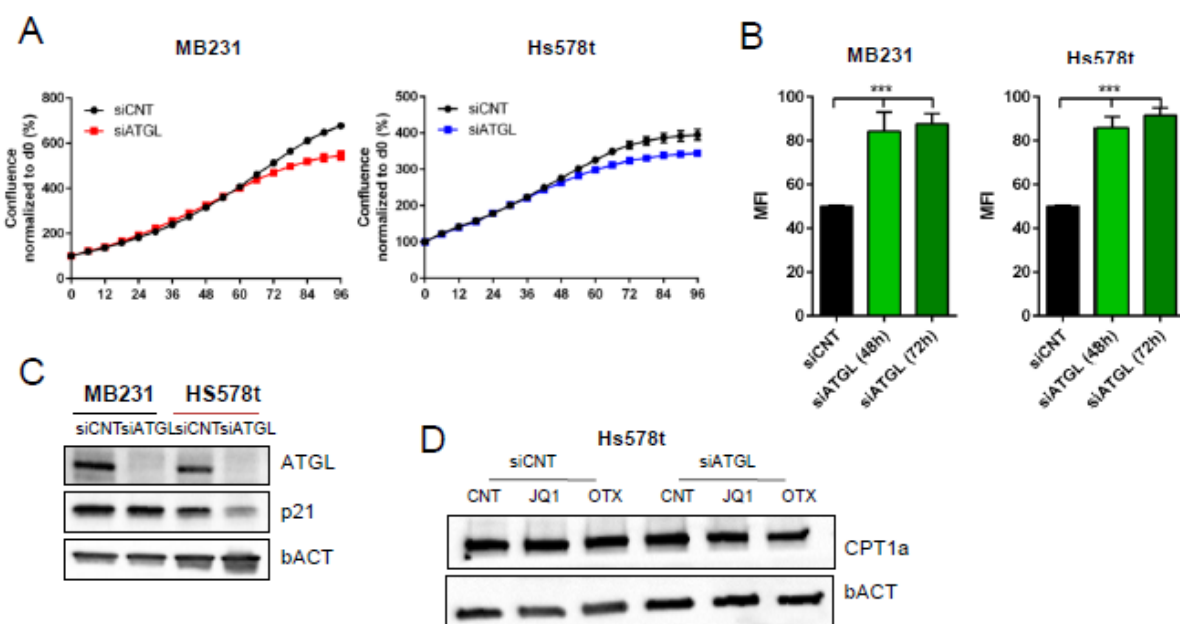

Supplementary figure 4

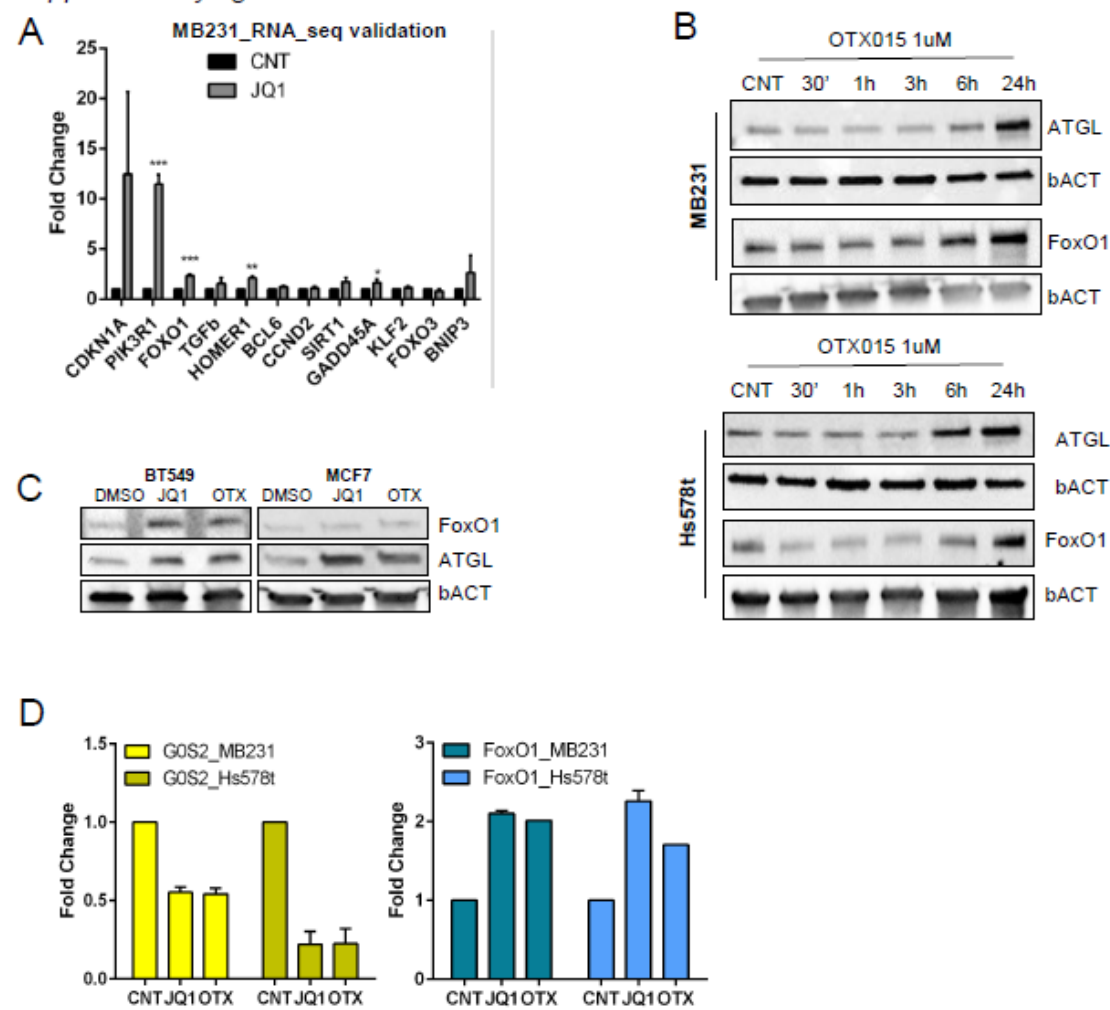

Supplement: Supplementary file 1 — Additional file 1: Supplementary Fig. 1. A. CFSE analysis of MDA-MB231 and HS578t after 24–48-72 hours of 1uM JQ1 treatment. Fluorescence decay is relative to the fluorescence at 24 hours of treatment B. RT-qPCR analysis of RNA-seq selected genes validation in Hs578t after 6 h treatment with JQ1 1 μM. C. Left, representative flow cytometry curves of BODIPY 500/510 fluorescence signal in MDA-MB 231 and Hs578t after 24 hours of 1 μM BETi treatment Right, flow cytometry analysis of LDs content stained with BODIPY 500/510 in MDA-MB 231 and Hs578t cell lines after 6 and 24 h of 1 μM BETi treatment (MFI = median fluorescence intensity). Supplementary Fig. 2. A. NMR analysis of metabolite content in Hs578t after 24 hours of 1 μM BETi treatment. B. Protein expression analysis of ATGL in all BC cell lines for 24 hours with 1 μM of BETi. C. Cell proliferation assay of MDA-MB231 and Hs578t measured by IncuCyte after adding different concentrations of FAs. OA: Oleic Acid; PA: Propionic Acid. D. CFSE analysis of MDA-MB231 and HS578t after 24–48-72 hours of 100uM and 200uM of oleic acid (OA, left) or 1 mM and 5 mM of propionate (PA, right) treatment. Fluorescence decay is relative to the fluorescence at 24 hours of treatment. E. Percentage of cell death in MDA-MB231 and Hs578t cells treated with 3 days with ATGListatin and stained with Annexin V. Representative images are selected for western blot. bACT served as a loading control. Supplementary Fig. 3. A. Cell proliferation assay of MDA-MB231 and Hs578t measured by IncuCyte after silencing ATGL. B. Flow cytometry evaluation of LDs content stained with BODIPY 500/510 in MDA-MB231 and Hs578t after 48 and 72 hours of siRNA transfection (siCNT vs siATGL). C. Immunoblotting for ATGL and p21 in MDA-MB231 and Hs578t cells after 48 hours of siRNA transfection (siCNT vs siATGL). D. Protein expression analysis of CPT1a after a combination of siRNA approach (siATGL for 48 h) and BETi treatment (1 μM for the last 24 hours) in Hs578t. Repres [file 13046_2022_2571_MOESM1_ESM.pdf]
